# Phonon‐Related Monochromatic THz Radiation and its Magneto‐Modulation in 2D Ferromagnetic Cr_2_Ge_2_Te_6_

**DOI:** 10.1002/advs.202103229

**Published:** 2021-10-29

**Authors:** Long Cheng, Huiping Li, Gaoting Lin, Jian Yan, Lei Zhang, Cheng Yang, Wei Tong, Zhuang Ren, Wang Zhu, Xin Cong, Jingjing Gao, Pingheng Tan, Xuan Luo, Yuping sun, Wenguang Zhu, Zhigao Sheng

**Affiliations:** ^1^ Anhui Key Laboratory of Condensed Matter Physics at Extreme Conditions High Magnetic Field Laboratory, HFIPS, Anhui Chinese Academy of Sciences Shushanhu Road 350 Hefei 230031 China; ^2^ ICQD Hefei National Laboratory for Physical Sciences at the Microscale and Key Laboratory of Strongly‐Coupled Quantum Matter Physics Chinese Academy of Sciences School of Physical Sciences University of Science and Technology of China No. 96, JinZhai Road, Baohe District Hefei Anhui 230026 China; ^3^ Key Laboratory of Materials Physics Institute of Solid State Physics HFIPS Chinese Academy of Sciences Shushanhu Road 350 Hefei Anhui 230031 China; ^4^ State Key Laboratory of Superlattices and Microstructures Institute of Semiconductors Chinese Academy of Sciences No. A35, QingHua East Road, Haidian District Beijing 100083 China; ^5^ Collaborative Innovation Center of Advanced Microstructures Nanjing University No. 22 Hankou Road, Gulou District Nanjing Jiangsu 210093 China

**Keywords:** magneto‐modulation, phonon‐polaritons, terahertz radiation, van der Waals materials

## Abstract

Searching multiple types of terahertz (THz) irradiation source is crucial for the THz technology. In addition to the conventional fermionic cases, bosonic quasi‐/particles also promise energy‐efficient THz wave emission. Here, by utilizing a 2D ferromagnetic Cr_2_Ge_2_Te_6_ crystal, first a phonon‐related magneto‐tunable monochromatic THz irradiation source is demonstrated. With a low‐photonic‐energy broadband THz pump, a strong THz irradiation with frequency ≈0.9 THz and bandwidth ≈0.25 THz can be generated and its conversion efficiency could even reach 2.1% at 160 K. Moreover, it is intriguing to find that such monochromatic THz irradiation can be efficiently modulated by external magnetic field below 160 K. According to both experimental and theoretical analyses, the emergent THz irradiation is identified as the emission from the phonon‐polariton and its temperature and magnetic field dependent behaviors confirm the large spin‐lattice coupling in this 2D ferromagnetic crystal. These observations provide a new route for the creation of tunable monochromatic THz source which may have great practical interests in future applications in photonic and spintronic devices.

## Graphical abstract

To addressing the challenge of low radiation efficiency of current universal terahertz sources, a van der Waals ferromagnetic semiconductor‐based efficient monochromatic terahertz radiation is developed here. It takes advantage of the bosonic quasi‐particles, phonon‐polaritons, that be coupled by exciting terahertz photons and inherent inter‐layered phonons in Cr_2_Ge_2_Te_6_. The radiation could also be effectively modulated by external magnetic fields.

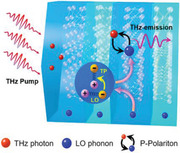

## 1 Introduction

Terahertz (THz) radiation source plays a crucial role in relevant research and applications. After decades of intensive cultivation, the playground of THz sources have been widely developed in many materials, such as semiconductors,^[^ ^1^ ^]^ nonlinear electro‐optic crystals,^[^ ^2^ ^]^ surface plasma,^[^ ^3^ ^]^ metamaterials,^[^ ^4^ ^]^ ferro‐/non‐magnetic heterojunctions,^[^ ^5^ ^]^ etc. Recently, with extraordinary electrical and optical properties, 2D van der Waals (vdWs) materials have been also used for the novel THz irradiation source development.^[^ ^6^ ^]^ For instance, Huang et al. reported the THz surface emission based on the competition between surface optical rectification and photocurrent surge in layered MoS_2_.^[^ ^7^ ^]^ Under the excitation of strong infrared pulses, Suo et al. proposed a new THz emitter based on the shift current occurring on the inversion‐broken surface of layered CrSiTe_3_ crystal.^[^ ^8^ ^]^ With the aid of laser‐excited surface plasmon on a gold substrate, Bahk et al. observed an enhanced THz emission from the single‐layered graphene.^[^ ^9^ ^]^ In the majority of these studies, THz radiation based on 2D vdWs materials are originating from photoconductive effect, optical rectification, and laser‐induced plasma, which all belong to the fermion (electron) catalog and generally bring broadband THz emission with low conversion efficiency.

In addition to the broadband cases, monochromatic THz sources are also important and it possesses immense potential in practical applications,^[^ ^10^ ^]^ especially in communication technology.^[^ ^11^ ^]^ According to the protocol of the International Telecommunication Union (ITU), the better monochromaticity of the waves in communication usually means higher band utilization and lower interference risk.^[^ ^12^ ^]^ From visible light to near‐infrared band, the monochromatic radiation sources (MRS) are usually realized by exciting the electronic transition between the direct bandgap of specific semiconductors.^[^ ^13^ ^]^ While to the extent of MRS in mid‐ and far‐ infrared range, the radiation sources would extend from fermionic particles to bosonic cases, such as phonons.^[^ ^14^ ^]^ For THz wave with lower photon energy, its MRS requires phonons with matched frequency, which usually could be observed in some artificial materials. For example, by designing proper semiconductor superlattice structures, people have observed the THz phonons and experimentally realized quasi‐monochromatic THz radiations.^[^ ^15^ ^]^

2D vdWs materials are natural superlattice/homojunction structures with weak inter‐layered interactions that are hundreds of times weaker than intra‐layered cases.^[^ ^16^ ^]^ Moreover, layered 2D vdWs materials possess not only high anisotropy but also rich variety of inter‐layered breathing phonon modes in THz range.^[^ ^17^ ^]^ This feature holds great potential for the monochromatic THz radiations from phonons in 2D vdWs materials. In comparison to fermionic (electron) mechanisms, due to the bosonic nature of phonons, large amount of phononic quasi‐/particles (e.g., phonons, phonon‐polaritons) could accumulate in a single condensate state.^[^ ^18^ ^]^ Corresponding radiation is independent of the phonon population and the population‐inversion in fermionic cases is not necessary.^[^ ^19^ ^]^ In addition, the energy is partially stored in the form of phonons with relatively long lifetime, which means the phonon‐related THz sources could operate with extremely small cavity sizes and maintain high quality factors.^[^ ^20^ ^]^ Moreover, the radiation frequency is mainly dominated by phonon modes, which usually possess narrow linewidth. This suggests a promising candidate of narrowband THz‐wave source.^[^ ^21^ ^]^ Thereby, the phonon related radiation mechanism in principle would possess the key performance indicators of “zero‐threshold”, high quality factors, and narrow linewidth.^[^ ^19^, ^22^ ^]^ Surprisingly, little attention has been devoted to the study of 2D vdWs bosonic THz wave and no cogenetic phonon‐based monochromatic THz radiation has been published yet.

Here, by choosing ferromagnet Cr_2_Ge_2_Te_6_ as a model system, we firstly demonstrate a 2D phonon‐based monochromatic THz radiation. It was found that a strong THz irradiation with frequency ≈0.9 THz and bandwidth ≈0.25 THz can be generated with a low‐photonic‐energy broadband THz pump. The conversion efficiency varies with temperature and can even reach 2.1% at 160 K. Moreover, it is intriguing to find that the external magnetic field (*B*) could efficiently modulate such monochromatic THz irradiation especially when *B* is parallel to the *ab*‐plane of the 2D Cr_2_Ge_2_Te_6_ crystal. After carefully experimental and theoretical examining, the emergent THz irradiation is identified as the emission from the phonon‐polariton in this 2D ferromagnetic crystal. Our findings suggest that the use of 2D materials may provide a variable source for bosonic‐related monochromatic THz radiation.

## 2 Results and Discussions

**Figure 1:**
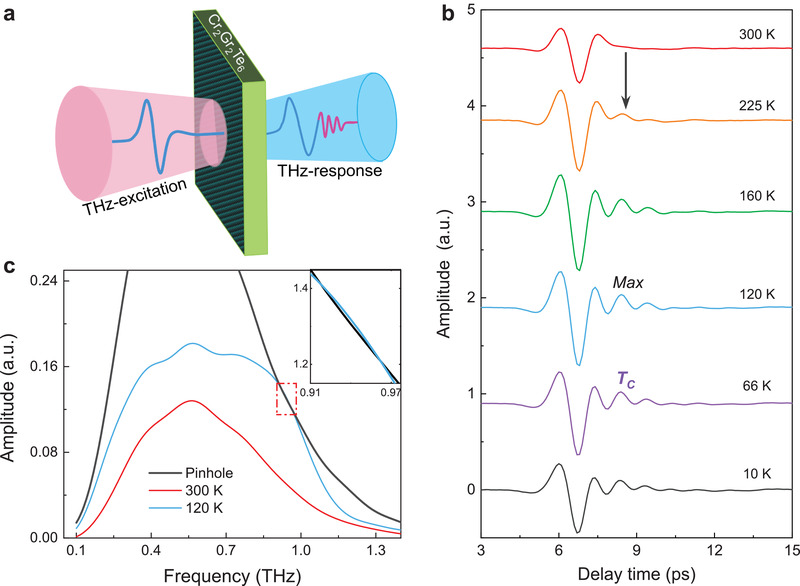
Terahertz responses of Cr_2_Ge_2_Te_6_ crystal. a) Schematic diagram of the transmission measurement configuration. b) Time‐resolved THz spectra transmitted through the sample at some critical temperatures. c) Frequency‐domain THz spectra corresponds to temperatures of 300 K (red curve) and 120 K (blue curve). The black curve is the reference THz signal through the pinhole of the sample holder without any sample. The inset is the close‐up view of the red dashed frame area at 120 K.

To create a phonon‐based THz emitter, appropriate materials are important. In addition to intensively studied metamaterial and graphene, 2D vdWs magnetic materials are another ideal material for this purpose because their exotic properties and their strong spin‐phonon coupling are expected to give effective responses to external magnetic fields.^[^ ^23^ ^]^ Cr_2_Ge_2_Te_6_ is one of the prototypes. It undergoes a paramagnetic‐ferromagnetic transition at *T*_C_ = 68 K.^[^ ^24^ ^]^ The THz transmission response of Cr_2_Ge_2_Te_6_ single crystal was measured by means of a home‐built terahertz time domain spectroscopy system (THz‐TDS) system as schematically shown in **Figure** **1**a. The measurements were taken from room temperature to 10 K with an Oxford Spectromag He‐bath cryostat (see details in Experimental Section). After penetrating through the sample, the time‐resolved THz signals were collected and the typical results are depicted in Figure 1b. In the time domains of transmitted THz wave obtained at 300 K, there is a main wave that has the same shape as the incident THz wave with lower intensity due to the absorption of Cr_2_Ge_2_Te_6_ crystal. With the temperature decreasing, the intensity of the main wave increases at first and then drops below 160 K. This could be attributed to the competition between attenuated absorption caused by semiconductor property and enhanced absorption due to the ferromagnetism during the cooling process (see more detail in Part I and Figure S1, Supporting Information).^[^ ^25^ ^]^ Apart from the main wave, it is intriguing to find that there are additional oscillations superimposed on the rear portion of the main waveforms (black arrow in Figure 1b). Such THz oscillations emerge at 225 K and exist until the lowest temperature of 10 K we measured. Its amplitude slightly varies with temperature and reaches the maximum at 120 K.

There are two possible origins for these additional oscillations. One is Fabry–Pérot effect that arises from multiple reflections between internal interfaces of the sample, another is electromagnetic (EM) radiations. The loss functions for both experimental results and theoretical predictions of Fabry–Pérot effect were calculated.^[^ ^26^ ^]^ As shown in the Figure S2 (Supporting Information), the experimental result is significantly different from the theoretical feature of the Fabry–Pérot effect. Especially, the loss function of these oscillations corresponds to the THz response around 0.9 THz and could even be negative, which indicates negative loss after penetrating the sample. In order to further explore the additional oscillations, the fast‐Fourier‐transformation from TDS results was done and the corresponding typical frequency domain spectra (FDS) results are depicted in Figure 1c. By comparing the results of 300 K (red curve) and 120 K (blue curve), it is found that the additional oscillations yield a certain frequency of around 0.9 THz. Especially, at 120 K, the amplitude of output THz wave at ≈0.9 THz is almost 2.7 times of the 300 K case and even higher than the free‐space pinhole reference (black curve), as shown in the inset of Figure 1c. Considering both the negative loss function and large output THz wave at ≈0.9 THz, it is reasonable to conclude that the additional oscillations are emergent THz radiation.

**Figure 2:**
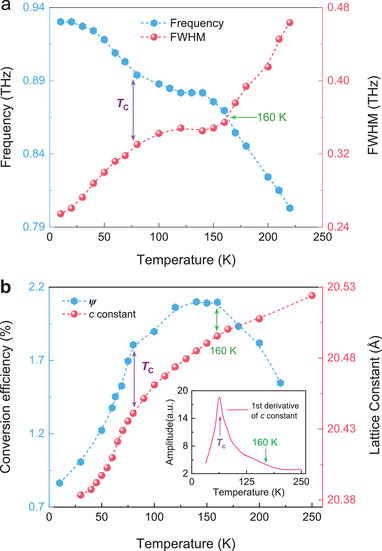
Temperature‐dependent properties of the radiation and inter‐layered lattice constant. a) Temperature‐dependent center frequency (*f*_c_, blue dashed line connected with hexagonal symbols) and FWHM (red dashed line marked with sphere symbols) of the radiation. b) Temperature dependence of optical‐to‐THz conversion efficiency (*ψ*) (blue dashed curve marked with hexagonal symbols) and lattice constant along *c*‐axis (sphere symbols connected with a red dashed curve). The purple and green arrows in a) and b) mark the transition temperatures around *T*_C_ and 160 K, respectively. The inset in (b) is the first derivative of the temperature‐dependent *c* constant.

Furthermore, by calculating the FDS of transmittance variation (*Delta T*) of specific temperature with reference to 300 K that without radiation emerged (Figure S3, Supporting Information), it is found that the additional oscillation is monochromatic and its center frequency, monochromaticity, and amplitude are strongly dependent on temperature. **Figure** **2**a presents the temperature dependence of the center frequency (*f*_c_) and full‐width at half‐maximum (FWHM) of the THz radiation. At first glance, the most important common performance is that the temperature dependence of *f*_c_ and FWHM separate the temperature coordinate into three regions with boundaries of 160 K and *T*_C_. While the striking contrast is that their dependencies are opposite. Specifically, for the case of *f*_c_, when the temperature drops from 220 to 160 K, it first dramatically increases from 0.80 to 0.87 THz. While in the following region from 160 K to *T*_C_, it turns to increase in a slowing trend from 0.87 to 0.90 THz. Afterward, at temperatures lower than *T*_C_, the frequency is further hardened to 0.93 THz. However, for the FWHM that indicates the monochromatism of the radiations, its temperature dependence in these three regions corresponds to the narrowing from 0.46 to 0.35 THz rapidly (220 to 160 K), the approximate plateau form from 0.35 to 0.32 THz (160 K to *T*_C_), and a further narrowing to 0.25 THz below *T*_C_, respectively.

As a rule of thumb, the conversion efficiency (*ψ*) is a critical characteristic.^[^ ^27^ ^]^ Here, the radiation performance is evaluated with the parameter of $\psi \;=\;I_{r}/I_{\mathrm{ab}}\;=\;E_{O}^{2}\;/\;\left(E_{i\;}^{2}-E_{t}^{2}\right)$, where *I*_ab_ and *I*_r_ are power (intensity) of absorbed pump pulse and generated THz radiation, in which the THz power is proportional to the square of its field amplitude. *E*_O_ is the P‐P amplitude of the first oscillation waveforms, *E*_i_ and *E*_t_ are P‐P_M_ of the incident and transmitted THz waveforms, respectively. Corresponding results are shown as hexagonal symbols in Figure 2b. The *ψ* also shows the same temperature regions as *f*_c_ and FWHM. From 220 to 160 K, the *ψ* starts to emerge and first be dramatically enhanced to ≈2.1%, which is higher than the cases of fermionic cases, such as photoconductive and nonlinear optical rectification (usually lower than 0.1%).^[^ ^28^ ^]^ Whereas in the region below 160 K, the increasing trend then gradually transforms into a declining one. Peculiarly, for temperatures below *T*_C_, it is dramatically attenuated and finally reaches ≈0.86% at 10 K. All in all, the temperature dependence of *ψ* possesses the same features and originations as the case of P‐P_M_, in which transformations below 160 K and *T*_C_ could attribute to the enhanced THz absorption arising from the formation of magnetic correlations.^[^ ^25^, ^29^ ^]^ However, the emergency temperature (225 K) of the THz radiation is far away from the Curie temperature (*T*_C_ ≈68 K) and also above the spin fluctuation temperature (≈160 K).^[^ ^25^, ^30^ ^]^ Moreover, the THz radiation frequency (≈0.9 THz/3.7 meV) of Cr_2_Ge_2_Te_6_ crystal is quite larger than its spin excitation gap (≈0.28 meV).^[^ ^31^ ^]^ These two factors imply that the observed THz radiation is not entirely spin‐related.

Previous studies on semiconductor‐based THz sources indicate that the THz radiations are usually assisted by intrinsic phonons in these materials.^[^ ^32^ ^]^ As shown above that the frequency, FWHM, and the intensity of the additional THz emission in this 2D material are temperature‐dependent and there are two critical temperatures (160 K and *T*_C_). The X‐ray diffraction (XRD) measurements with temperature variation were done and the temperature‐dependent lattice constant along *c*‐axis is shown in Figure 2b (red spheres). It is found that the lattice constant along *c*‐axis between two CGT layers decreases with the decrease of the temperature and the decreasing slopes change twice when the sample was cooling from room temperature down to 25 K. As shown by its first‐order derivative curve in the inset of Figure 2b, the critical temperatures corresponding to the decreasing slope variations are 160 K and *T*_C_, which are same as those for the parameters of the THz emission. Such coincidence implies that the observed THz emission is associated with the inter‐layered phonons in this 2D vdWs ferromagnetic material.

**Figure 3:**
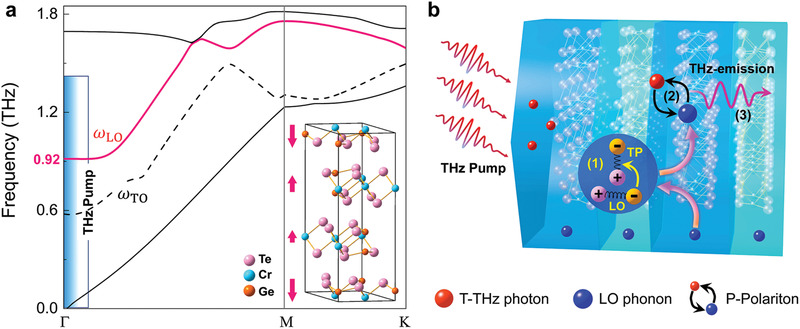
Schematic of phonon‐polariton generating THz radiation scheme. a) Sketched phonon dispersion of Cr_2_Ge_2_Te_6_ crystal corresponding to the THz radiation. The THz radiation involved zone‐folded LO layer‐breathing mode is depicted with red curve. The black dashed curve is the inter‐layered transverse optical (TO) phonon mode. The shaded region exhibits the spectral range of incident THz pump. Inset shows the crystal structure of the Cr_2_Ge_2_Te_6_ crystal, in which these red arrows indicate the oscillation directions of the inter‐layered breathing mode. b) Diagram of the coupling of LO layer‐breathing mode and THz photons, resulting in the formation of P‐Polaritons and ensuing monochromatic THz EM emission. The inset marked with a pink arrow illustrates the intermediate (TP) during the whole process. The formation processes of TP, P‐Polariton, and THz emission correspond to (1), (2), and (3), respectively.

Owing to the unit cell of the Cr_2_Ge_2_Te_6_ expanded along *c*‐axis, the Brillouin zone is folded in the momentum space, resulting in the emergence of zone‐folded phonon modes. Based on the XRD results, we calculated the phonon dispersion of the expanded hexagonal cell (see detailed results in Figure S4, Supporting Information). As notably shown with red arrows in the inset of **Figure** **3**a, there is an inter‐layered longitudinal vibration mode, corresponding to a non‐centrosymmetric longitudinal optical (LO) layer‐breathing mode with frequency (*ω*_LO_) of ≈0.92 THz at Γ point. Such LO mode with the three layers within each unit cell possesses different vibration amplitudes (red curve in Figure 3a). It should be emphasized that this LO layer‐breathing mode, which breaks the inversion center and degenerates the space group from $R\bar{3}$ to *R*3, is dipole active and gives rise to piezoelectricity.^[^ ^33^ ^]^ Interestingly, due to the piezoelectricity and generalized Hooke's law of the updated group, some co‐frequency transverse vibrations with transversal polarization (TP) can be driven by the vibration of LO phonons, as schematically shown with the (1) of Figure 3b. According to the Lyddane‐Sachs‐Teller (LST) relation,^[^ ^16a^ ^]^ after pumping by a THz pulse envelope with transversal polarized electric field, the incident EM waves with frequency close to the LO layer‐breathing mode could effectively propagate in Cr_2_Ge_2_Te_6_ and strongly coupled with the co‐frequency TP and form a new bosonic quasi‐particle, i.e., so‐called phonon‐polariton (P‐Polariton).^[^ ^34^ ^]^ After that, these photon pumping induced bosonic P‐Polariton could in turn generate far‐field EM radiation with frequency close to the original LO phonon as schematically illustrated by process (2) and (3) in Figure 3b.^[^ ^14^, ^35^ ^]^ While for the transverse optical (TO) phonon mode, there exist certain absorption and reflection loss during the propagating process. The TO mode also happens to be at the peak of the frequency spectrum of the THz system. There is neither distinct loss effect nor TO phonon involved radiation effect observed. Moreover, due to the energy of this bosonic P‐Polariton generated THz radiation is carried in the piezoelectricity vibrations, it is reasonable to consider it as a highly efficient bosonic laser‐like process.^[^ ^20^, ^36^ ^]^ Beyond that, according to the further calculations (Figure S5, Supporting Information), it was found that the *c* constant dependent evolution of phonon frequency is consistent with both the experimental results of *f*_c_ shown in Figure 2a and the previous reports of interactions between spin and dipole‐active phonon mode in 2D magnets.^[^ ^23^, ^37^ ^]^

According to the above discussion and the tensor symmetry of *R*3 structure, it can be predicted that, for a certain THz‐Pump field, the electric displacement and corresponding THz radiation should possess a 6‐fold in‐plane symmetry (see Part VI of the Supporting Information). In order to confirm this issue, the in‐plane anisotropic dependence of the THz radiation amplitude was investigated by tuning the in‐plane azimuth of the sample. As shown in Figure S6b (Supporting Information), it exhibits a cycle of *π*/3 that matches well with the 6‐fold symmetry of the P‐Polariton as predicted. In addition, the THz excitation amplitude dependence of the radiation amplitude was also investigated by inserting two THz polarizers after the low‐temperature‐grown GaAs photoconductive antenna (LT‐GaAs‐PCA). As shown in Figure S6d (Supporting Information), the radiation and excitation amplitudes are linearly correlated and there is no saturation and threshold observed, which is also in good consistent with the feature of bosonic lasing reported before.^[^ ^18^ ^]^ These features not only indicate that the radiation effect could be further improved by enhancing the stimulation intensity, but also means that the stimulated irradiation source in Cr_2_Ge_2_Te_6_ is extremely sensitive to the weak THz field (≈kV cm^−1^). This is in good accordance with the extremely high sensitivity of Cr_2_Ge_2_Te_6_ to external low‐intensity photonic field.^[^ ^38^ ^]^ Unlike other THz sources based on 2D materials,^[^ ^7^, ^8^, ^9^ ^]^ this unique THz‐MRS takes full advantage of the inter‐layered breathing phonons and achieves highly efficient monochromatic THz radiations with stimulation of low‐energy THz pulse. This mechanism is of great practical interests, such as monochromatic THz laser, integrated gain‐type monochromatic THz filter, key relay components in photonic communication, etc.

Different from other 2D semiconductors, Cr_2_Ge_2_Te_6_ holds ferromagnetism. Except for electron and phonon, spin is non‐negligible degree and it has been demonstrated that there exist strong spin‐phonon interactions in Cr_2_Ge_2_Te_6_ and its isostructural Cr_2_Si_2_Te_6_ materials.^[^ ^23^, ^39^ ^]^ As presented in Figure 2, it is found that not only the *c*‐axis lattice constant but also the amplitude, frequency, and FWHM of the emergent THz emission have abnormal near around the *T*_C_ of Cr_2_Ge_2_Te_6_ crystal, which also implies the subtle interplays between the phonon‐involved THz radiation and spin orders. Hence, it is reasonable to expect that the THz radiation could also be tuned by external magnetic field (*B*).

**Figure 4:**
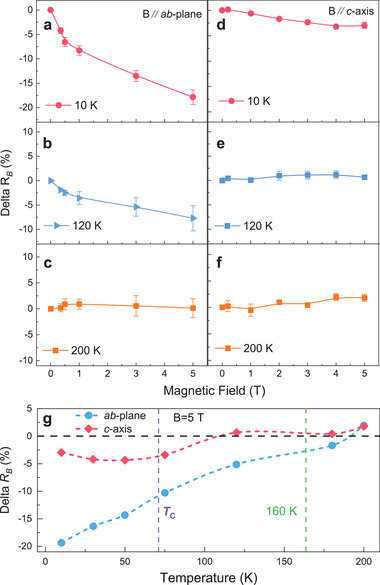
Magnetic field and temperature‐dependent modulation effect of THz radiation. a–f) At 10, 120, and 200 K, *B* dependence of *Delta R*_B_ at corresponding *f*_c_ with *B* (from 0 to 5 T) applied along *c*‐axis and *ab*‐plane, respectively. Error bars in a‐f) represent uncertainties in determining the *Delta R*_B_. g) Temperature‐dependent *Delta R*_B_ under a uniform *B* of 5 T. Blue and red curves marked with solid circle and square symbols are *Delta* *R_B_* with *B* applied along *ab*‐plane and *c*‐axis, respectively. The purple and green dashed lines are boundaries of the temperature regions.

The effect of *B* on the THz radiations was investigated by the THz‐TDS system with a superconductor magnet. The system stability under high magnetic field was verified beforehand (see Figure S7a, Supporting Information). At low temperatures, there is significant variation of the THz response that could be observed when *B* is applied (see Figure S7b–d (Supporting Information). In order to present the magnetic field effect on the THz emission, the relative THz amplitude variation *Delta R*_B_ was defined as *Delta R*_B_ = (*E*_B –_ *E*_B0_)/*E*_B0_, in which *E*_B_ and *E*_B0_ are THz responses of Cr_2_Ge_2_Te_6_ crystal with and without *B* applied. The magnetic field dependence of *Delta R*_B_ obtained at corresponding *f*_c_ and different temperatures were summarized in **Figure** **4**a–f. For *B*//*c*‐axis, the *Delta R*_B_ is negligible above *T*_C_ and it is also small (< −5%) below *T*_C_. When *B*//*ab*‐plane, the *Delta R*_B_ starts to emerge around 160 K and increases with decreasing of the temperature. Notably, at 10 K, the *Delta R*_B_ in *ab*‐plane could reach the maximum of around −20% (with *B* = 5 T), which is greater than the case of *c*‐axis (≈−3%). Such anisotropic feature of the magnetic field modulation effect on *Delta R*_B_ can be clearly observed in Figure 4g, in which the temperature‐dependent *Delta R*_B_ under *B* = 5 T is plotted. By applying an external magnetic field, the spin state of the Cr_2_Ge_2_Te_6_ crystal can be modified for both long‐range order (below *T*_C_) and short‐range order (*T*_C_ <*T*<160 K).^[^ ^25^, ^30^ ^]^ As one of the possible mechanisms, the magnetic field induced the spin‐flips scattering could suppress the electron‐phonon interactions as well as the dipole active phonon involved P‐Polariton.^[^ ^40^ ^]^ Accordingly, with such large spin‐phonon coupling in this 2D ferromagnetic material, the switching of spin state by *B* would bring the modulation of phonon‐related THz emission. Moreover, the spontaneous spin orientation of Cr_2_Ge_2_Te_6_ is along *c*‐axis. And then, from viewpoint of spin modulation, the magnetic field effect is significant when *B*//*ab*‐plane. While for the case of *B*//*c*‐axis, the spin orientation is not modified much under the codirectional magnetic field and the *Delta R*_B_ does not seem to be perturbed as much as the *B*//*ab*‐plane case. These are consistent with our observations shown in Figure 4. The magneto modulation effect provides another proof for its P‐Polariton‐related radiation mechanism. All these features not only offer another degree of freedom for the regulation of THz radiation in practical applications, but also provide a piece of convincing evidence for the correlation between the THz radiation, phonons, and spin orders.

## 3 Conclusion

In summary, benefiting from the coherent LO layer‐breathing mode, a magneto‐tunable monochromatic THz radiation was demonstrated in a 2D vdWs ferromagnet Cr_2_Ge_2_Te_6_. After pumping by a broadband THz wave, a strong monochromatic THz irradiation can be generated. The frequency, FWHM, and the intensity of the emergent THz radiation vary with the temperature and its conversion efficiency could even reach 2.1% at 160 K. Our experimental and theoretical analyses indicate that the emergent THz irradiation is from the pump induced phonon‐polariton in this 2D vdWs material. Moreover, due to the existing strong spin‐phonon coupling, it is interesting to find that such monochromatic THz irradiation can be efficiently modulated by the magnetic field below 160 K. In addition to the general fermionic cases, our findings suggest that the use of 2D vdWs ferromagnet might provide a viable source for the realization of bosonic THz source with tunable and monochromatic features, which may have great practical interests in future applications in photonic and spintronic devices.

## 4 Experimental Section

The Cr_2_Ge_2_Te_6_ single crystals are prepared with the self‐flux technique, following the procedure described in ref. ^[^ ^41^ ^]^. The effective size of the sample is ≈6×4×0.2 mm. The terahertz data was collected with a home‐built terahertz time domain spectroscopy system (THz‐TDS) has been introduced in detail in ref. ^[^ ^42^ ^]^. The sample is assembled on the sample holder of Oxford Instruments Spectromag He‐bath cryostat, with which one could realize test environments of temperature from 10 to 300 K and magnetic field up to 7 Tesla. After propagating through these fused silica windows of the cryostat, the coverage of the THz spectral is from 0.1 to 1.5 THz. The temperature‐dependent X‐ray diffraction (XRD) was measured with the XRD setup using high‐intensity graphite monochromatized Cu K*α* radiation system (Rigaku corporation, Japan). Then, the influence of the cooling process on the copper base is excluded and finally obtain the accurate temperature‐dependent evolution of the lattice constant.

### Ab Initio Calculations

First‐principles calculations based on density functional theory (DFT) were performed using the Vienna ab‐initio simulation package (VASP)^[^ ^43^ ^]^ with the projector‐augmented wave (PAW)^[^ ^44^ ^]^ pseudopotentials and the Perdew–Burke–Enzerhof^[^ ^45^ ^]^ parameterization of the generalized gradient approximation (GGA). The kinetic energy cutoff was set as 400 eV. A Γ‐centered 8×8×3 Monkhorst‐Pack grid was used for k‐point sampling. van der Waals corrections were treated by the semi‐empirical DFT‐D3 method.^[^ ^46^ ^]^ Optimized atomic structures were achieved until the forces on each atom were smaller than 0.01 eV Å^−1^. The phonon band structures were calculated with the frozen‐phonon finite difference method using the phonopy^[^ ^47^ ^]^ code interfaced with VASP.

## Conflict of Interest

The authors declare no conflict of interest.

## Supporting information

Supporting Information

## Data Availability

The data that support the findings of this study are available from the corresponding author upon reasonable request.
