# Supplementary material for: Phonon‐Related Monochromatic THz Radiation and its Magneto‐Modulation in 2D Ferromagnetic Cr2Ge2Te6
Source: Adv Sci (Weinh). 2021 Oct 29;9(1):2103229. doi: 10.1002/advs.202103229 (PMC8728850; doi:10.1002/advs.202103229)
Supplement: Supplementary file 1 — Supporting Information [file ADVS-9-2103229-s001.pdf]

© 2021 Wiley-VCH GmbH

ADVANCED SCIENCE
Open Access

Supporting Information

for *Adv. Sci.,* DOI: 10.1002/advs.202103229

Phonon-related monochromatic THz radiation and its magneto-modulation in 2D ferromagnetic $Cr_2Ge_2Te_6$

Long Cheng, Huiping Li, Gaoting Lin, Jian Yan, Lei Zhang, Cheng Yang, Wei Tong, Zhuang Ren, Wang Zhu, Xin Cong, Jingjing Gao, Pingheng Tan, Xuan Luo, Yuping sun, Wenguang Zhu*, Zhigao Sheng**

Supporting Information

Phonon-related monochromatic THz radiation and its magneto-modulation in 2D ferromagnetic $Cr_2Ge_2Te_6$

Long Cheng, Huiping Li, Gaoting Lin, Jian Yan, Lei Zhang, Cheng Yang, Wei Tong, Zhuang Ren, Wang Zhu, Xin Cong, Jingjing Gao, Pingheng Tan, Xuan Luo, Yuping sun, Wenguang Zhu*, Zhigao Sheng**

Contents:

I. **Temperature dependence of P-P_M**

II. **System stability verification at same temperature (*T*) and loss function of sample**

III. **Frequency domain spectra of *Delta T***

IV. **Calculation results of phonon dispersion**

V. **Estimation of longitudinal optical (LO) phonon**

VI. **In-plane anisotropy and stimulation dependence**

VII. **System stability verification under external magnetic field (B) and *B* modulation of THz response**

I. Temperature dependence of P-P_M

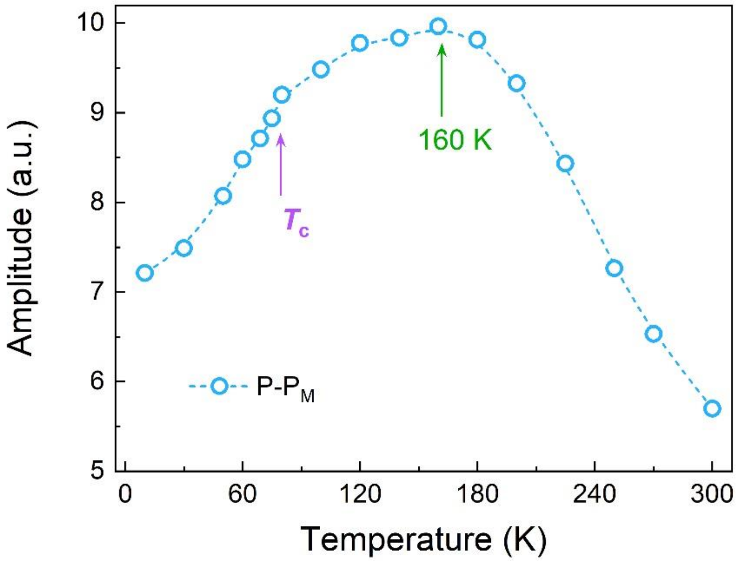

Figure S1 Temperature dependent peak-to-peak amplitude of the transmitted THz main waveforms (P-P_M).

According to the time-resolved spectra of the transmitted THz signals, the peak-to-peak amplitude of the main waveforms (P-P_M) could be easily picked out. Its temperature dependence is depicted in Figure S1. The evolution of the P-P_M divided the temperature coordinate into three regions with the boundaries of 160 K and T_C. To be specific, for the main waveforms in time domain, as the temperature drops from 300 K, the P-P_M was significantly enhanced until 160 K. Afterwards, along with the temperature further dropped in the range of 160 to 120 K, the upward trend of the P-P_M slows down and reaches the maximum. Peculiarly, the P-P_M enhancement of 120 K with respect to the 300 K case could reach ~40%. At last, the trend eventually develops into a steep declining one below T_C and reaches the minimum at 10 K. According to previous studies on THz responses of semiconductor materials, decrease of temperature could reduce the concentration of intrinsic carriers. Therefore, the enhancement of

transmittance at temperature from 300 K to 160 K could be attributed to the attenuated transition of intrinsic carriers among intra-band levels.[1] As to the temperature below 160 K, magnetic correlations start to appear and ensuing spin-electron interactions come into play, which dramatically give rise to an absorption in THz range.[2] As a result, during the cooling process, along with the enhancement of spin correlations, the competition of these two mechanisms would subsequently bring about the transition and decline of the P-P_M amplitude.

II. System stability verification at same temperature (T) and loss function of the sample

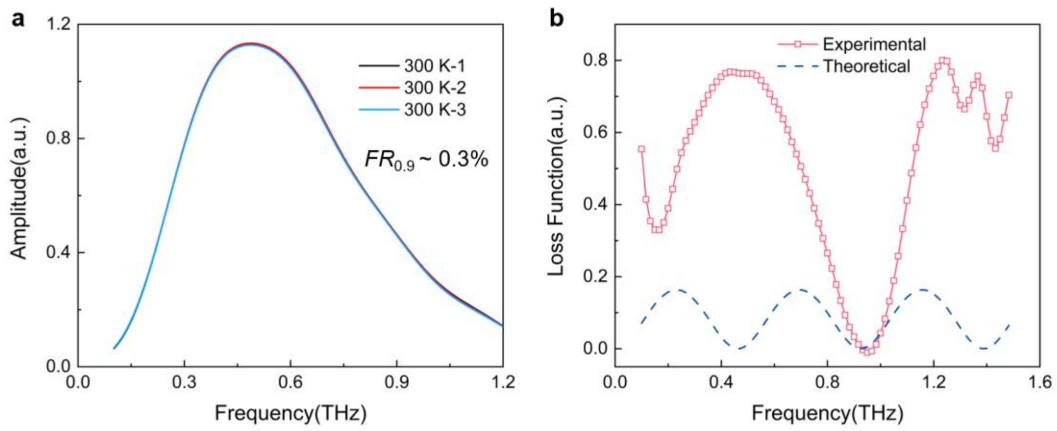

Figure S2 a) System stability verification at 300 K with multiple measurements. The maximal fluctuation rate at ~0.9 THz ($FR_{0.9}$) is calculated following the formula: $FR_{0.9} = \mathrm{Max}\{[E_{0.9}(\mathrm{T})\text{-}E_{0.9}(\mathrm{reference})]/E_{0.9}(\mathrm{reference})\}$, in which the reference is the 300 K-1 case. The $FR_{0.9}$ is almost zero (~0.3%) and the system is barely fluctuated at a certain temperature. b) Loss function of experimental (red square) and theoretical (blue dashed) results at 120 K. All these results are calculated according to ref. [3] The experimental results merely correspond to the THz response at ~0.9 THz, which is significantly different from the characteristic of Fabry–Pérot (FP) effect that originates from multiple reflections of the THz pulse between the internal interface of the sample (shown as dashed curve). Especially, the loss function of these oscillations corresponds to the frequency around 0.9 THz and could even be negative at 120 K, which indicates negative loss after penetrating the sample.

III. Frequency domain spectra of *Delta T*

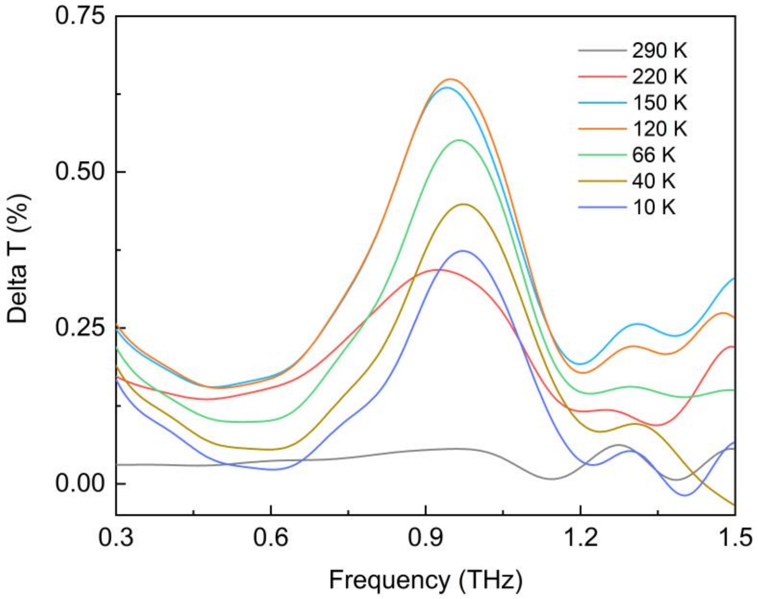

Figure S3 At specific temperature, frequency domain spectra (FDS) of the transmittance variation (*Delta T*) with reference to the case of 300 K. The results are calculated according to the formula of *Delta T*=(E_T-E_0)/E_r, where the E_T and E_0 are FDS of the sample at specific and room temperature, respectively. While E_r is the FDS of THz pulse penetrate through the pinhole of the sample holder without $Cr_2Ge_2Te_6$ mounted. This processing method can make the performance of the radiation effect in FDS more intuitive and significant.

IV. Calculation results of phonon dispersion

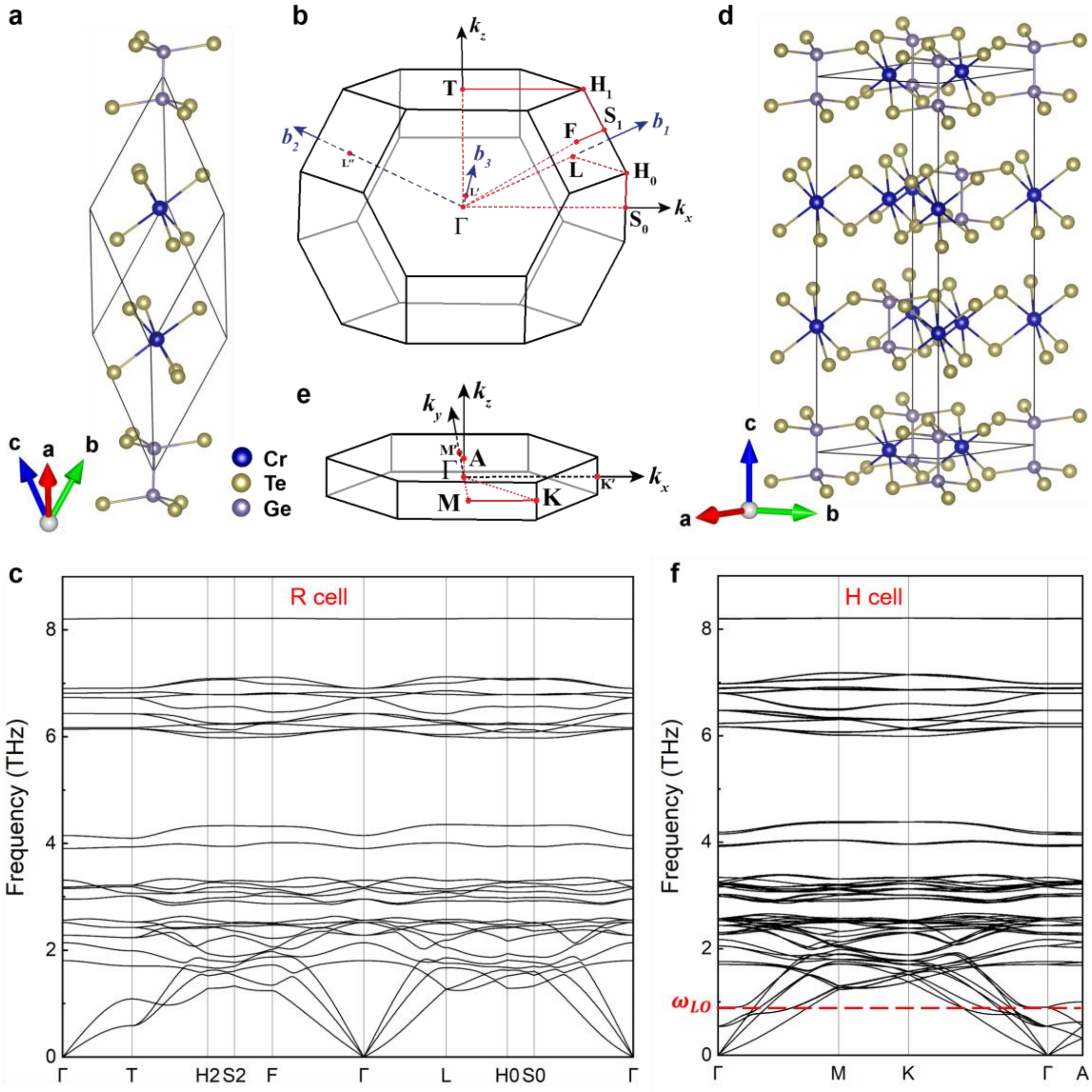

Figure S4 The crystal structure, Brillouin zone, and phonon dispersion of $Cr_2Ge_2Te_6$ crystal. a) The rhombohedral primitive cell (R cell) of $Cr_2Ge_2Te_6$. Blue, purple, and golden spheres represent Cr, Ge, and Te atoms, respectively. b) The Brillouin zone corresponds to the R cell. c) Phonon dispersion of the R cell. d) Crystal structure of $Cr_2Ge_2Te_6$ in a hexagonal conventional cell (H cell). e) Brillouin zone of the H cell. f) Zone-folded phonon dispersion of the H cell. The LO layer-breathing mode with frequency (ω_{LO}) around 0.92 THz is marked with red dashed line.

According to the inter-layered lattice constant derived from XRD results, the crystal structure of $Cr_2Ge_2Te_6$ is elongated along *c*-axis.[4] Consequently, the unit cell of $Cr_2Ge_2Te_6$ would transform from rhombohedral primitive cell (R cell) to hexagonal cell

(H cell), which induces the folding of the Brillouin zone and emergence of zone-folded phonon mode in the momentum space.[5] Corresponding the phonon dispersion of original R cell and the expanded H cell were calculated, as shown in Figure S5.

V. Estimation of longitudinal optical (LO) phonon

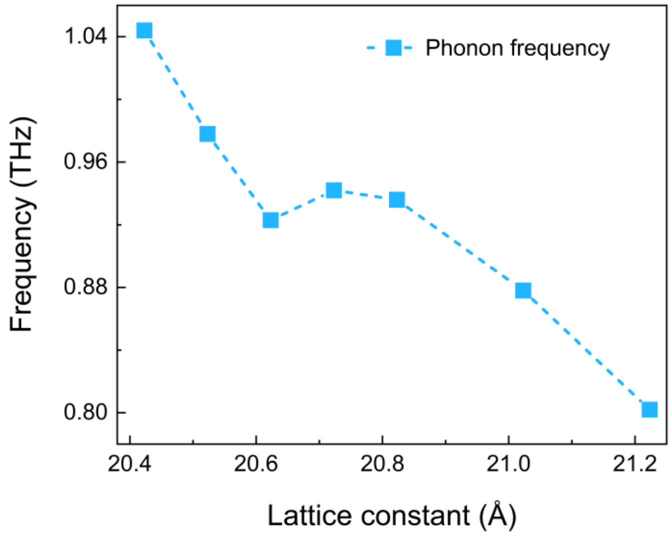

Figure S5 Estimation of the spin-phonon interaction induced frequency evolution of the inter-layered longitudinal optical (LO) phonon mode with respect to the lattice constant.

VI. In-plane anisotropy and stimulation dependence

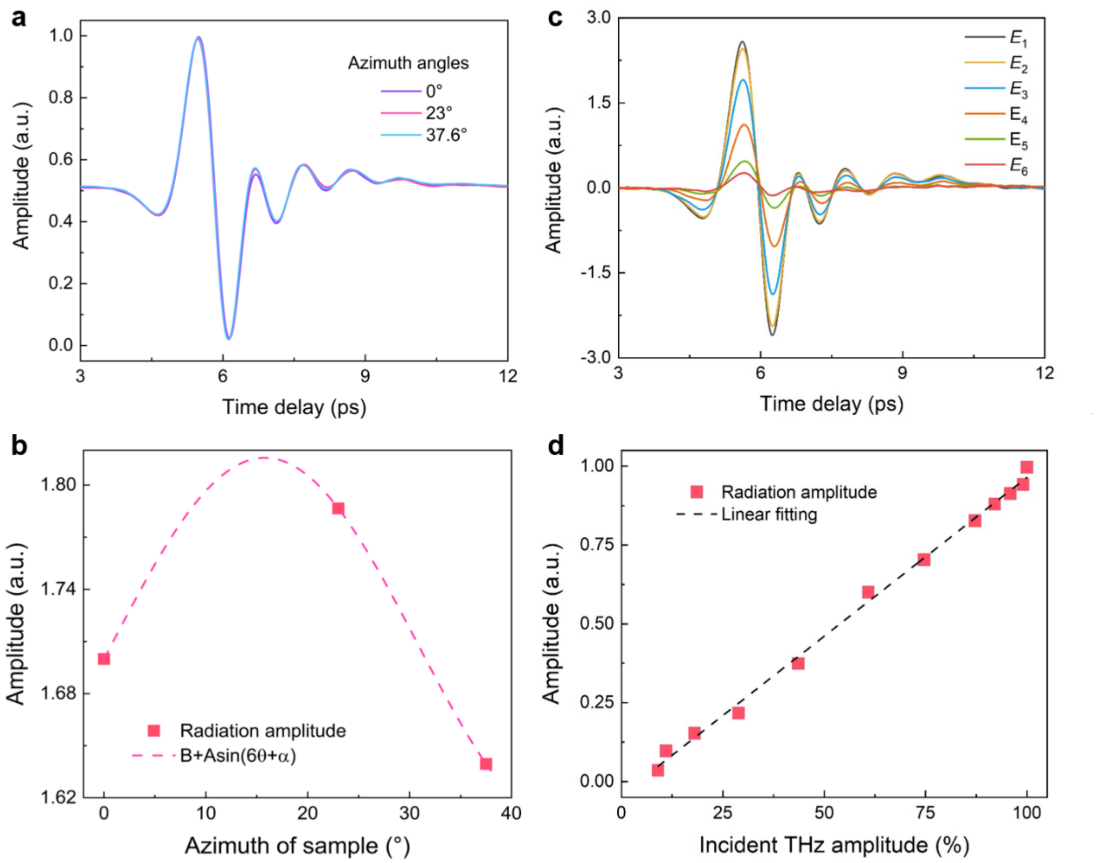

Figure S6 In-plane azimuth and incident THz amplitude dependence of the radiation effect at 120 K. a) Normalized time-resolved spectra of $Cr_2Ge_2Te_6$ with in-plane azimuth of the sample rotated from zero to 37.6°. b) Sample azimuth dependence of the radiation amplitude. These red dashed line in (b) is sinusoidal fitting results for in-plane anisotropy. c) Time resolved spectral of $Cr_2Ge_2Te_6$ with incident THz amplitude of the main waveforms decreased from E_1 to E_6. All these E_n are P-P amplitude of the incident THz pulse. d) Incident THz amplitude dependence of radiation amplitude. These percentages of x-coordinate are defined with E_n/E_1. The dashed line is the linear fitting result.

In this part, the in-plane anisotropy and incident THz amplitude dependence of the radiation effect is investigated by performing the in-plane azimuth dependence. For the case of variating azimuth of the sample, due to the degenerated crystal point group of $R3$, it's sufficient to choose three azimuth angles within the symmetry period ($2\pi/3$) to verify the in-plane anisotropy. As shown in Figure S6a and b, for these three different

in-plane azimuth angles, they also demonstrate certain azimuth dependence. The sinusoidal fitting result yields the 6-fold symmetry that twice of the crystal case. While for the incident THz amplitude dependence of the radiation effect, the radiation amplitude is linearly dependent with respect to the amplitude of the incident THz-pump field.

Given the non-centrosymmetric dipole-active LO phonon mode of the $Cr_2Ge_2Te_6$ crystal, its piezoelectric (PE) performance under external electric field could be expressed as:

$$S_i = s_{ij}\sigma_j + d'_{ij}E_j \tag{S1}$$

$$D_i = d_{ij}\sigma_j + \varepsilon_{ij}E_j \tag{S2}$$

where the s_{ij}, d_{ij}, d'_{ij}, S_i, σ_j, and ε_{ij} are simplified tensors of compliance, direct PE effect, converse PE effect, phonon involved strain and stress, and permittivity, respectively. While D_i and E_j are vectors of electric displacement and electric field.[6] Specifically, for the case of degenerated $R3$ group, ε_{ij}, d_{ij}, and s_{ij} for $Cr_2Ge_2Te_6$ could be expressed as:[7]

$$\boldsymbol{\varepsilon_{ij}} = \begin{bmatrix} \varepsilon_{11} & 0 & 0 \\ 0 & \varepsilon_{11} & 0 \\ 0 & 0 & \varepsilon_{33} \end{bmatrix} \tag{S3}$$

$$d_{ij} = \begin{bmatrix} d_{11} & -d_{11} & 0 & d_{14} & d_{15} & -d_{22} \\ -d_{22} & d_{22} & 0 & d_{15} & -d_{14} & -d_{11} \\ d_{31} & d_{31} & d_{33} & 0 & 0 & 0 \end{bmatrix} \tag{S4}$$

$$s_{ij} = \begin{bmatrix} s_{11} & s_{12} & s_{13} & s_{14} & -s_{25} & 0 \\ s_{12} & s_{11} & s_{13} & -s_{14} & s_{25} & 0 \\ s_{13} & s_{13} & s_{33} & 0 & 0 & 0 \\ s_{14} & -s_{14} & 0 & s_{44} & 0 & s_{25} \\ -s_{25} & s_{25} & 0 & 0 & s_{44} & s_{14} \\ 0 & 0 & 0 & s_{25} & s_{14} & \frac{1}{2}(s_{11} - s_{12}) \end{bmatrix} \quad \text{(S5)}$$

Then, according to previous studies, the components of the compliance tensor possess the values in the same magnitude of $s_{11} = 14.1 \times 10^{-12}\ \mathrm{Pa}^{-1}$ and $s_{13} = -5.1 \times 10^{-12}\ \mathrm{Pa}^{-1}$, whose difference is much smaller than other 2D materials.[8] As a result, the mechanical vibration of the LO phonon could result in considerable co-frequency transverse strain ($S_1 = s_{13}\sigma_3$) and corresponding piezoelectric displacement ($\mathrm{D}_{PE} = d_{11}\sigma_1$). Moreover, by coupling with the co-frequency part of incident transversal envelop of THz-pump field (E_1), they could be further coherently enhanced and yield stronger transversal vibrations ($S_1 = s_{13}\sigma_3 + d'_{11}E_1$) and electric displacement ($D_1 = \mathrm{D}_{PE} + D_{THz-pump} = d_{11}\sigma_1 + \varepsilon_{11}E_1$), which is also the formation process of a new bosonic particle, namely phonon-polariton (P-Polariton). In addition, from the equation of Eq.(S2), under a certain THz-Pump field, we can find the electric displacement is proportional to the product of d_{ij} and σ_j. Accordingly, the 6-fold symmetry shown in Figure S4d could be attributed to the 3-fold of the PE tensor (d_{ij}) multiplied by the 2-fold of the phonon involved stress (σ_j). While for a certain azimuth angle and phonon mode, the linear dependence of the radiation on the incident THz amplitude corresponds to the second term on the right side, which indicates $D_i \propto \varepsilon_{ij}E_j$.

VII. System stability verification under *B* and *B* modulation of THz response

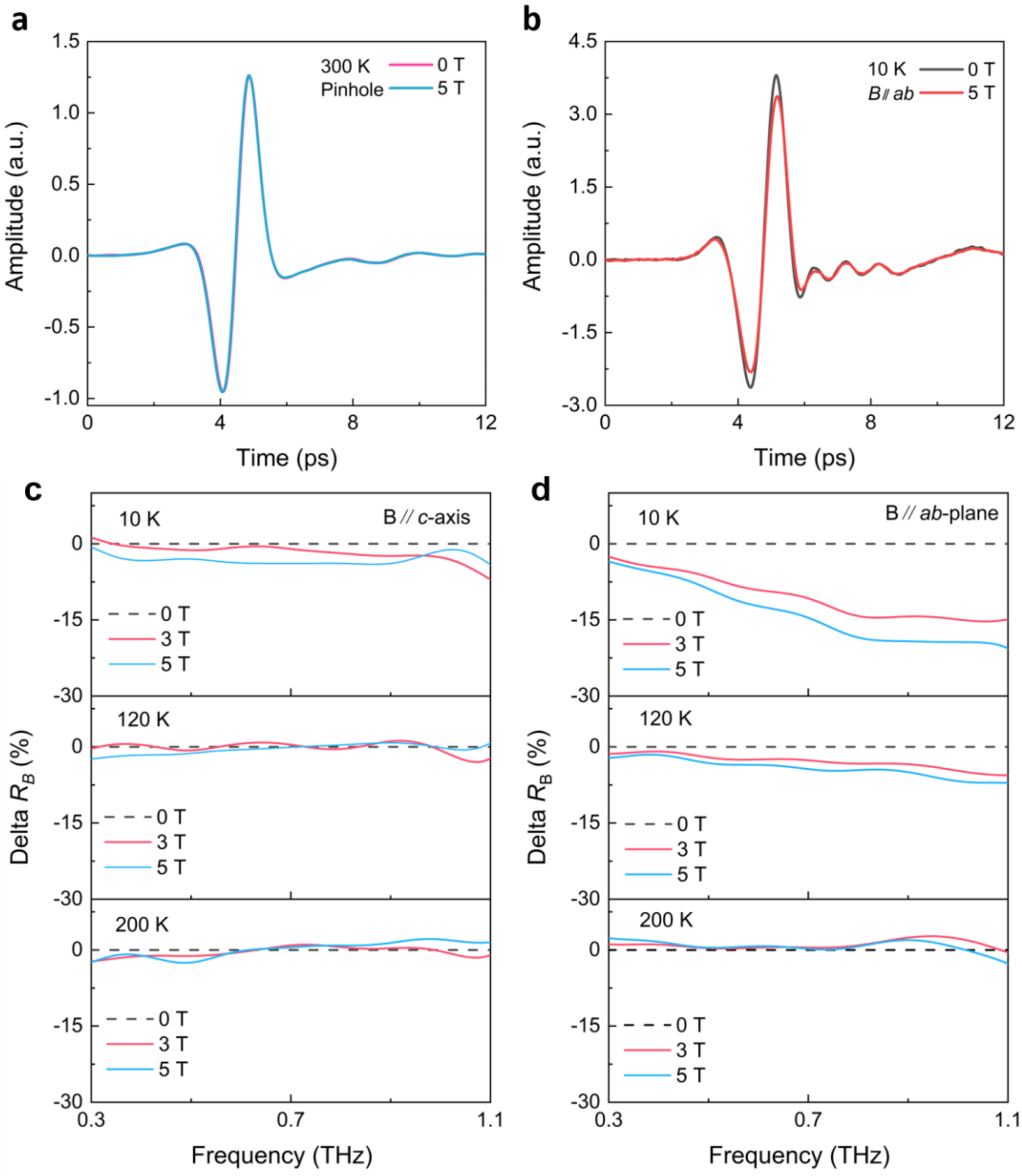

Figure S7 System stability verification and terahertz response of $Cr_2Ge_2Te_6$ under external magnetic field (***B***). a) The red and blue curves are the time-resolved waveforms penetrating the pinhole of sample holder without and with magnetic field of 5 T applied. The verification was performed at 300 K. b) At 10 K, Time resolved THz responses of $Cr_2Ge_2Te_6$ without and with ***B*** of 5 T applied along the *ab*-plane. c) At 10 K, 120 K, and 200 K, frequency domain spectral of *Delta* R_B with 0 T, 3 T, and 5 T magnetic fields applied along the *c*-axis. d) Frequency domain spectral of *Delta* R_B with magnetic field along *ab*-plane and temperature same as (c). The ***B*** comes to affect the amplitude of the radiation, especially in the case along *ab*-plane. While no obvious shift of f_c was observed.

Reference

[1] a) R. Ulbricht, E. Hendry, J. Shan, T. F. Heinz, M. Bonn, *Rev. Mod. Phys.* **2011**, *83* (2), 543; b) M. Kira, W. Hoyer, S. W. Koch, *Phys. Status Solidi B* **2003**, *238* (3), 443.

[2] a) A. Pinczuk, B. S. Dennis, D. Heiman, C. Kallin, L. Brey, C. Tejedor, S. Schmitt-Rink, L. N. Pfeiffer, K. W. West, *Phys. Rev. Lett.* **1992**, *68* (24), 3623; b) V. Gudmundsson, J. J. Palacios, *Phys. Rev. B* **1995**, *52* (15), 11266; c) Y. Sun, W. Tong, X. Luo, *Phys. Chem. Chem. Phys.* **2019**, *21* (45), 25220.

[3] R. V. Mikhaylovskiy, E. Hendry, V. V. Kruglyak, R. V. Pisarev, T. Rasing, A. V. Kimel, *Phys. Rev. B* **2014**, *90* (18), 184405.

[4] B. Siberchicot, S. Jobic, V. Carteaux, P. Gressier, G. Ouvrard, *J. Phys. Chem.* **1996**, *100* (14), 5863.

[5] C. Kittel, *Introduction to Solid State Physics*, Wiley, **2004**.

[6] a) Y. Y. Zhu, X. J. Zhang, Y. Q. Lu, Y. F. Chen, S. N. Zhu, N. B. Ming, *Phys. Rev. Lett.* **2003**, *90* (5), 053903; b) X. Zhang, R. Zhu, J. Zhao, Y. Chen, Y. Zhu, *Phys. Rev. B* **2004**, *69* (8), 085118; c) J. Yang, *An Introduction to the Theory of Piezoelectricity*, Springer US, **2006**.

[7] R. E. Newnham, *Properties of Materials: Anisotropy, Symmetry, Structure*, OUP Oxford, **2004**.

[8] M. de Jong, W. Chen, T. Angsten, A. Jain, R. Notestine, A. Gamst, M. Sluiter, C. Krishna Ande, S. van der Zwaag, J. J. Plata, C. Toher, S. Curtarolo, G. Ceder, K. A. Persson, M. Asta, *Sci. Data* **2015**, *2* (1), 150009.
